# Dr. William A. McCorn

**Published:** 1904-04

**Authors:** 


Dr. William A. McCorn, of Long Island City, N. Y., University of Buffalo, 1882, died February 18, 1904.

